# Re-Irradiation in Patients with Recurrent Rectal Cancer is Safe and Feasible

**DOI:** 10.1245/s10434-021-10070-6

**Published:** 2021-05-22

**Authors:** Esmée A. Dijkstra, Véronique E. M. Mul, Patrick H. J. Hemmer, Klaas Havenga, Geke A. P. Hospers, Christina T. Muijs, Boudewijn van Etten

**Affiliations:** 1 Department of Medical Oncology, University Medical Centre Groningen, University of Groningen, Groningen, The Netherlands; 2 Department of Radiation Oncology, University Medical Centre Groningen, University of Groningen, Groningen, The Netherlands; 3 Department of Surgery, University Medical Centre Groningen, University of Groningen, Groningen, The Netherlands

## Abstract

**Background:**

There is no consensus yet for the best treatment regimen in patients with recurrent rectal cancer (RRC). This study aims to evaluate toxicity and oncological outcomes after re-irradiation in patients with RRC in our center. Clinical (cCR) and pathological complete response (pCR) rates and radicality were also studied.

**Methods:**

Between January 2010 and December 2018, 61 locally advanced RRC patients were treated and analyzed retrospectively. Patients received radiotherapy at a dose of 30.0–30.6 Gy (reCRT) or 50.0–50.4 Gy chemoradiotherapy (CRT) in cases of no prior irradiation because of low-risk primary rectal cancer. In both groups, patients received capecitabine concomitantly.

**Results:**

In total, 60 patients received the prescribed neoadjuvant (chemo)radiotherapy followed by surgery, 35 patients (58.3%) in the reRCT group and 25 patients (41.7%) in the long-course CRT group. There were no significant differences in overall survival (*p* = 0.82), disease-free survival (*p* = 0.63), and local recurrence-free survival (*p* = 0.17) between the groups. Patients in the long-course CRT group reported more skin toxicity after radiotherapy (*p* = 0.040). No differences were observed in late toxicity. In the long-course CRT group, a significantly higher cCR rate was observed (*p* = 0.029); however, there was no difference in the pCR rate (*p* = 0.66).

**Conclusions:**

The treatment of RRC patients with re-irradiation is comparable to treatment with long-course CRT regarding toxicity and oncological outcomes. In the reCRT group, less cCR was observed, although there was no difference in pCR. The findings in this study suggest that it is safe and feasible to re-irradiate RRC patients.

**Supplementary Information:**

The online version contains supplementary material available at 10.1245/s10434-021-10070-6.

Despite the improved treatment of primary rectal cancer, recurrent rectal cancer (RRC) remains a problem. After long-course neoadjuvant chemoradiotherapy (CRT) followed by total mesorectal excision (TME) for locally advanced rectal cancer (LARC), RRC is seen in 5–9% of patients.^1^–^3^ Intermediate-risk primary tumors [cT1-3N1, cT3N0 with > 5 mm extramural vascular invasion (EMVI) or distant to the mesorectal fascia (MRF) > 1 mm] are treated with 5×5 Gy radiotherapy followed by TME. In intermediate rectal cancer patients, the risk of RRC is approximately 5%.^4^ Even in low-risk rectal cancer patients (cT1-2N0 or cT3N0 with ≤ 5 mm EMVI, MRF > 1 mm) in whom neoadjuvant radiotherapy is omitted, there is still a 4–6% chance of RRC.^5^^,^^6^

In cases of intermediate primary rectal cancer or LARC, patients receive (chemo)radiotherapy. If RRC occurs in these prior irradiated patients, the neoadjuvant re-irradiation dose is limited^7^ by the risk of potential normal tissue complications.^8^ Nonetheless, is re-irradiation with a lower dose still effective? In the literature, there is no consensus for the best treatment regimen in patients with RRC.^9^–^11^ Re-irradiation doses range from 15.0 to 49.2 Gy and 30.0 to 30.6 Gy, and median doses of 40.8 Gy.^12^–^14^ A study has been conducted that determines the radiotherapy dose on the basis of retreatment interval,^15^ and the systematic review by Tanis et al. demonstrated that there are studies providing adjuvant radiotherapy in the case of RRC. Furthermore, chemotherapy was not always used as a radiosensitizer.^10^

In RRC, just as in LARC, neoadjuvant CRT could be used to downstage and downsize the tumor, resulting in a better chance of an R0 resection. However, resection of RRC is more difficult because of the altered and varied anatomy of organs and critical structures in the pelvis as a result of the initial treatment. Furthermore, differences in tumor growth and the presence of post-treatment fibrosis make the resection more challenging. Therefore, the risk of an R1 resection is substantial,^16^–^18^ resulting in worse survival.^11^ To obtain free resection margins (R0), an extensive (i.e. multivisceral) resection procedure must often be performed.^19^–^22^

This study aimed to evaluate toxicity and the oncological outcome of low-dose re-irradiation and concurrent chemotherapy, compared with high-dose primary radiotherapy and concurrent chemotherapy, in RRC patients. Furthermore, radicality, clinical complete response (cCR) and pathological complete response (pCR) rates after neoadjuvant treatment were evaluated.

## Methods

Overall, 61 consecutive patients with clinically resectable locally advanced RRC without distant metastasis during staging and who received neoadjuvant (chemo)radiotherapy between January 2010 and December 2018 were retrospectively evaluated. This study was conducted in accordance with the Declaration of Helsinki. Our Institutional Review Committee approved this analysis and waived informed consent because of the retrospective study design.

Local recurrent disease was defined as clinically and/or histopathologically proven recurrent disease within the pelvis. Staging was performed using (diffusion-weighted imaging) magnetic resonance imaging (MRI), computed tomography (CT) scan, and colonoscopy with biopsies if possible. All patients were then discussed in a multidisciplinary rectal cancer board meeting to determine the best treatment strategy. According to Kusters et al., the tumor location was classified into the following subsites: lateral (pelvic sidewall, immediately behind the posterior ischiac spine, in the obturator lymph node compartment, or along the iliac vessels), presacral (predominantly midline, in contact with the sacral bone), anterior (predominantly midline, involving the bladder, uterus, vagina, seminal vesicles, or prostate), anastomosis (after low anterior or low Hartmann, at the staple line), and perineal (perineum, anal sphincter complex with surrounding perianal and ischiorectal space).^23^

Patients who previously received radiotherapy for their primary tumor were re-irradiated with 30.0–30.6 Gy (2.0–1.8 Gy/fraction daily) using a three- or four-field technique, and received concurrent capecitabine 825 mg/m^2^ twice daily (on working days). The second group of radiotherapy-naïve patients were irradiated with 50.0–50.4 Gy (2.0–1.8 Gy/fraction daily) using a three- or four-field technique, and also received concurrent capecitabine 825 mg/m^2^ twice daily (on working days). In both groups, the radiotherapy target volume was tumor with margin, the mesorectal area, and presacral and internal iliac lymph node regions.^24^ Approximately 6 weeks after neoadjuvant treatment, patients were restaged and were then discussed in the multidisciplinary board to determine the clinical response and resection strategy.

Surgery was planned 8–12 weeks after the completion of CRT. The following resections were performed: low anterior resection, abdominoperineal resection, partial pelvic exenteration, total pelvic exenteration, abdominosacral resection, and other type of resection (not organically bound). In the case of a potential irradical resection (R1), intraoperative brachytherapy (IOBT) was scheduled. Frozen sections were not mandatory to determine if IOBT should be performed. A flexible intraoperative template (FIT) was used to cover the irradical area, while 1 × 10 Gy was applied at 1 cm of the FIT.

All specimens were fixed in formalin for at least 24 h. Resection margin status was classified as follows: R0 resection [free margins (> 1 mm) ], R1 resection [microscopically involved margins (≤ 1 mm)], and R2 resection (macroscopically involved margins).^25^ pCR defines the absence of residual tumor in the totally embedded resection specimen.

During and after neoadjuvant treatment, outpatient visits were scheduled to check the well-being of the patient. Any physical complaints during radiotherapy and chemotherapy were reported in the patients’ file by the radiotherapist and medical oncologist, respectively. In retrospect, we graded these symptoms according to the Common Terminology Criteria for Adverse Events (CTCAE) version 5.^26^ Acute postoperative toxicity within 30 days after surgery and late postoperative toxicity within 90 days after surgery were reported in the medical file by the surgeon. We graded these symptoms in retrospect according to the Clavien–Dindo classification^27^ for acute toxicity, and CTCAE version 5^26^ for late toxicity. Follow-up was routinely performed with yearly CT scanning of the thorax and abdomen, regular carcinoembryonic antigen (CEA) testing, and outpatient visits.

### Statistical Analysis

Statistical analyses were performed using SPSS statistical software version 23 for Windows (IBM Corporation, Armonk, NY, USA). Proportions were compared using Chi-square tests, and continuous parameters, depending on the distribution of the data, were compared using a *t* test or Mann–Whitney *U* test. A two-sided *p* value of < 0.05 was considered statistically significant. The sensitivity of MRI-based cCR was calculated as the percentage of the number of cCRs on MRI divided by the number of pCRs at histopathological evaluation, while the specificity of MRI-based cCR was calculated as the percentage of the number of non-cCRs on MRI divided by the number of non-pCRs at histopathological evaluation. Sensitivity of the radicality of the resection and frozen sections was calculated as the percentage of the number of R0 resections during surgery or on frozen sections divided by the number of histopathological R0 resections. The specificity was calculated as the percentage of the number of R1 resections during surgery or as a result of the frozen section, divided by the number of R1 resections at histopathological evaluation. Median follow-up was calculated using the reverse Kaplan–Meier method. Overall survival (OS) was calculated from the date of resection until the last follow-up or death by all causes; disease-free survival was calculated from the date of resection until the date of recurrence (local and/or distant), last follow-up, or death by all causes; and local recurrence-free survival (LRFS) was calculated from the date of resection until the date of local recurrence, last follow-up, or death by all causes. OS, DFS, and LRFS were calculated using the Kaplan–Meier method and were tested using the log-rank test.

## Results

Between January 2010 and December 2018, 61 patients were diagnosed with locally advanced RRC without distant metastasis at staging (35 reCRT and 26 radiotherapy-naïve patients). All primary tumor and patient characteristics are shown in Table 1. Reasons why patients in the long-course CRT group did not receive neoadjuvant radiotherapy for the primary tumor are summarized in Table 1. Other reasons for no neoadjuvant therapy in the long-course CRT group were double tumor in the colon and rectum (*n* = 3), previous prostate carcinoma requiring radiotherapy (*n* = 1), adenocarcinoma accidentally found (*n* = 5), and unknown (*n* = 1). In Table 2, the RRC tumor and patient characteristics are shown. The median interval between primary tumor resection and diagnosis of RRC was 25 months [interquartile range (IQR) 19–48] in the reCRT group (*n* = 35) and 20 months (IQR 13–41) in the long-course CRT group (*n* = 26). In 85.2% of patients, the RRC was preoperatively histologically proven. Reasons why the RRC was not preoperatively histologically proven were: not able to perform a biopsy (*n* = 2), negative biopsy result with a strong suspicion of recurrent disease (*n* = 4), and not performed but strongly suspected recurrence (*n* = 3).

**Table 1:** Patient and treatment characteristics of the primary rectal tumor

	ReCRT [*n* = 35]		Long-course CRT [*n* = 26]		*p* value
Sex					0.52
Male	20	(57.1)	17	(65.4)	
Female	15	(42.9)	9	(34.6)	
Age, years (median [IQR])	62	[52–69]	68	[63–73]	0.030
Tumor stage					0.027
cT1-2N0	7	(20.0)	10	(38.5)	
cT1-2N +	1	(2.9)	1	(3.8)	
cT3-4N0	10	(28.6)	6	(23.1)	
cT3-4N +	17	(48.6)	5	(19.2)	
Unknown	–		4	(15.4)	
Type of neoadjuvant treatment					–
50.0/50.4 Gy with chemotherapy	19	(54.1)			
25 Gy	15	(42.9)			
Prematurely stopped^a^	1	(2.9)			
Reason no neoadjuvant treatment					–
cT1-2N0 tumor			10	(38.5)	
Rectosigmoid carcinoma			4	(15.4)	
High proximal rectal tumor			2	(7.7)	
Other			10	(38.5)	
Type of resection					0.016
LAR	13	(37.1)	15	(57.7)	
APR	20	(57.1)	4	(15.4)	
TEM	1	(2.9)	4	(15.4)	
Hartmann	1	(2.9)	–		
Total exenteration	–		1	(3.8)	
Other	–		2	(7.7)	
Definite pathology resection					0.37
R0	26	(74.3)	20	(76.9)	
R1	8	(22.9)	3	(11.5)	
R2	–	–	1	(3.8)	
Unknown	1	(2.9)	2	(7.7)	
Histology tumor at histopathological evaluation					0.39
Adenocarcinoma	34	(97.1)	26	(100.0)	
Mucinous	1	(2.9)	–	–	

Data are expressed as *n* (%) unless otherwise specified

*IQR* interquartile range, *CRT* chemoradiotherapy, *LAR* low anterior resection, *APR* abdominoperineal resection, *TEM* transanal endoscopic microsurgery, *R0* clear resection margins, *R1* resection margin ≤ 1 mm, *R2* macroscopic residual tumor

^a^Received 46.8 Gy due to radiation proctitis with severe pain

**Table 2:** Patient characteristics of the recurrent rectal tumor

	ReCRT [*n* = 35]		Long-course CRT [*n* = 26]		*p* value
Sex					0.52
Male	20	(57.1)	17	(65.4)	
Female	15	(42.9)	9	(34.6)	
Age, years (median [IQR])	65	[53–72]	70	[64–75]	0.030
Histology tumor (preoperative)					0.10
Adenocarcinoma	26	(74.3)	26	(100.0)	
Negative result biopsy	4	(11.4)	–		
No biopsy taken	5	(14.3)	–		
Location^23^					0.025
Lateral	12	(34.3)	4	(15.4)	
Presacral	4	(11.4)	3	(11.5)	
Anterior	10	(28.6)	2	(7.7)	
Anastomosis	6	(17.1)	13	(50.0)	
Perineum	3	(8.6)	4	(15.4)	
Tumor stage					0.027
cT1-2N +	1	(2.9)	1	(3.8)	
cT3-4N0	28	(80.0)	14	(53.8)	
cT3-4N +	6	(17.1)	10	(38.5)	
cTx^a^ N0	–		1	(3.8)	

Data are expressed as *n* (%) unless otherwise specified

*CRT* chemoradiotherapy, *IQR* interquartile range

^a^Tumor could not be assessed

The location of the locally advanced RRC was mostly lateral in the reCRT group (34.3%) and at the site of the anastomosis in the long-course CRT group (50.0%). Although not significant, lateral recurrence occurred about twice as often in the reCRT group (34.3% vs. 15.4%, *p* = 0.10). Overall, there was a significant difference in tumor location (*p* = 0.025).

In the reCRT group (*n* = 35), all patients received radiotherapy as scheduled. Chemotherapy was omitted in one patient (2.9%) because of the severe prior adverse effects of capecitabine (severe nausea, vomiting, and diarrhea) and two patients (5.7%) prematurely stopped chemotherapy because of severe diarrhea (*n* = 1) and coronary spasm (*n* = 1).

In the long-course CRT group (*n* = 26), one patient (3.8%) received 5 × 5 Gy radiotherapy only. In addition, chemotherapy was omitted in this patient because of age (80 years) and comorbidities. This patient had less extensive neoadjuvant treatment and was therefore excluded from further analysis, leaving 25 patients in the long-course CRT group, all of whom were treated with radiotherapy and received concurrent chemotherapy (Table 3). One patient (4.0%) prematurely stopped chemotherapy because of thrombopenia.

**Table 3:** Neoadjuvant treatment and surgical characteristics of the recurrent rectal tumor

	ReCRT [*n* = 35]		Long-course CRT [*n* = 25]		*p* value
ReCRT group					
30.0/30.6 Gy without chemotherapy	1	(2.9)			
30.0/30.6 Gy with chemotherapy	34	(97.1)			
Completed n(C)RT	32	(94.1)			
Long-course CRT group					
50.0/50.4 Gy with chemotherapy			25	(100.0)	
Completed n(C)RT			24	(96.0)	
cCR					
Yes	1	(2.9)	5	(20.0)	0.029
Partial	20	(57.1)	13	(52.0)	0.69
No	12	(34.3)	7	(28.0)	0.61
Tumor growth	2	(5.7)	–		0.22
Type of resection					0.24
LAR	–		3	(12.0)	0.035
APR	11	(31.4)	12	(48.0)	0.19
Partial exenteration	3	(8.6)	–		0.13
Total exenteration	9	(25.7)	5	(20.0)	0.61
ASR	1	(2.9)	1	(4.0)	0.81
Other (not organically bound)	11	(31.4)	4	(12.0)	0.17
IOBT					0.046
No	21	(60.0)	21	(84.0)	
Yes	14	(40.0)	4	(16.0)	
Radicality of resection by PA					0.11
R0	15	(42.9)	17	(68.0)	0.05
R1	18	(51.4)	6	(24.0)	0.033
R2	–		1	(4.0)	0.23
Irresectable	2	(5.7)	1	(4.0)	0.76
pCR					0.66
Yes	3	(8.6)	3	(12.0)	
No	32	(91.4)	22	(88.0)	
Histology tumor at histopathological evaluation					0.45
Adenocarcinoma	30	(85.7)	22	(88.0)	0.80
Mucinous	2	(5.7)	–		0.22
No rest tumor (pCR)	3	(8.6)	3	(12.0)	0.66
Tumor differentiation grade					0.029
Well	8	(22.9)	9	(36.0)	0.27
Well–moderately	5	(14.3)	9	(36.0)	0.050
Moderately	15	(42.9)	1	(4.0)	0.001
Poorly	2	(5.7)	2	(8.0)	0.73
Irresectable	2	(5.7)	1	(4.0)	0.76
pCR	3	(8.6)	3	(12.0)	0.66

Data are expressed as *n* (%)

*CRT* chemoradiotherapy, *n(C)RT* neoadjuvant (chemo)radiotherapy, *cCR* clinical complete response, *LAR* low anterior resection, *APR* abdominoperineal reaction, *ASR* abdominosacral resection, *IOBT* intraoperative brachytherapy, *PA* pathology, *R0* clear resection margins, *R1* resection margin ≤1 mm, *R2* macroscopic residual tumor, *pCR* pathological complete response

A cCR was seen on MRI imaging in one (2.9%) and five patients (20.0%) in the reCRT and long-course CRT groups, respectively (*p* = 0.029) (Table 3). Sensitivity and specificity of MRI-based cCR were 33.3% and 100% in the reCRT group and 66.6% and 86.4% in the long-course CRT group, respectively.

Surgical characteristics are shown in Table 3. Every patient underwent surgery (*n* = 60) and the median time between the end of neoadjuvant treatment and surgery was 11 weeks (IQR 9–14) in the reCRT group and 13 weeks (IQR 10–15) in the long-course CRT group. The type of resection in the reCRT group was more than twice as often not organ-specific, but not significant (*p* = 0.17). Frozen sections were taken in only 18 patients (16 in the reCRT group and 2 in the long-course CRT group). The sensitivity and specificity of frozen sections in the total patient group were 85.7% and 72.7%, respectively.

IOBT was performed significantly more often in the reCRT group (14 vs. 4, *p* = 0.046). Overall, an R1 resection was suspected perioperatively in 18 patients. All but one patient received IOBT; in that patient, IOBT was omitted because of a negative frozen section (R0). Once IOBT was performed in a patient in whom it was judged that an R2 resection was accomplished, the frozen section however demonstrated an R1 resection. Overall, 4 of 18 patients (22.2%) were overtreated with IOBT (R0 resection and IOBT performed). In all patients in the long-course CRT group in whom the surgeon judged the resection was R1, IOBT was performed; at histopathological evaluation, 50% of these resections were R1. The accuracy of intraoperative judgment of radicality of resection is accompanied by a sensitivity and specificity of 80.0% (12 perioperative R0/15 pathological R0) and 64.7% (11 perioperative R1/17 pathological R1) in the reCRT group, and 88.9% (16 perioperative R0/18 pathological R0) and 33.3% (2 perioperative R1/6 pathological R1) in the long-course CRT group, respectively.

Histopathologically proven R0 resections were accomplished in 42.9% and 68.0% of patients in the reCRT group and long-course CRT group, respectively (*p* = 0.05). R1 resections were seen in 51.4% of reCRT patients and 24.0% of long-course CRT patients (*p* = 0.033). Overall, 5.7% of patients in the reCRT group were irresectable. In the long-course CRT group, 4% of patients were irresectable, and in 4% of patients an R2 resection was accomplished. There were no significant differences in pCR (8.6% and 12.0% in the reCRT and long-course CRT groups, respectively; *p* = 0.66) (Table 3).

No differences were observed in the number of grade I–II (*p* = 0.48) and grade III (*p* = 0.76) tumors, and no grade IV or V toxicities were reported (Table 4). Only two patients in the reCRT group experienced grade III toxicity after radiotherapy (*n* = 1, obstruction) and chemotherapy (*n* = 1, diarrhea). Patients who were treated with long-course CRT reported skin toxicities significantly more often (*p* = 0.040); no differences were observed in chemotherapy-related toxicity. In regard to surgery, there were no significant differences in acute or late postoperative toxicity (Table 4), and there was no difference between neoadjuvant-related toxicity and interval until tumor recurrence (< 1 year or ≥ 1 year between surgery of the primary tumor and the start of neoadjuvant treatment of RRC; *p* = 0.80).

**Table 4:** Toxicity related to neoadjuvant chemoradiotherapy and surgery

	ReCRT [*n* = 35]		Long-course CRT [*n* = 25]		*p* value
Patients who reported toxicity after nCRT (any grade)	17	(48.6)	14	(56.0)	0.57
Highest grade adverse event reported per patient (CTCAE)					
Grade I–II	15	(42.9)	13	(52.0)	0.48
Grade III	2	(5.7)	1	(4.0)	0.76
Toxicity related to radiotherapy (CTCAE)					
Gastrointestinal toxicity	8	(22.9)	7	(28.0)	0.65
Nervous system toxicity	2	(5.7)	1	(4.0)	0.76
Skin toxicity	2	(5.7)	6	(24.0)	0.040
Urinary toxicity	–		1	(4.0)	0.23
Toxicity related to chemotherapy (CTCAE)					
Blood toxicity	1	(2.9)	1	(4.0)	0.81
Cardiac toxicity	1	(2.9)	–		0.39
Gastrointestinal toxicity	4	(11.4)	2	(8.0)	0.66
Nervous system toxicity	–		1	(4.0)	0.23
Skin toxicity	3	(8.6)	–		0.13
Patients who reported toxicity after surgery (any grade)	29	(82.9)	19	(76.0)	0.57
Highest grade adverse event reported per patient (CD/CTCAE)					
Grade I–II	20	(57.1)	14	(56.0)	0.93
Grade III	9	(25.7)	5	(20.0)	0.61
Acute toxicity (CD)					
Gastrointestinal toxicity	7	(20.0)	6	(24.0)	0.71
Infection	8	(22.9)	6	(24.0)	0.92
Neurological toxicity	15	(42.9)	9	(36.0)	0.59
Sexual toxicity	1	(2.9)	–		0.39
Renal toxicity	9	(25.7)	8	(32.0)	0.59
Wound healing disorder	10	(28.6)	5	(20.0)	0.45
Late toxicity (CTCAE)					
Infection	2	(5.7)	–		0.22
Insufficient fracture	1	(2.9)	–		0.39
Neurological toxicity	6	(17.1)	5	(20.0)	0.78
Renal toxicity	1	(2.9)	–		0.39
Wound healing disorder	–		1	(4.0)	0.23

Data are expressed as *n* (%)

*CRT* chemoradiotherapy, *nCRT* neoadjuvant chemoradiotherapy, *CTCAE* Common Terminology Criteria for Adverse Events, *CD* Clavien–Dindo

**Fig. 1:**
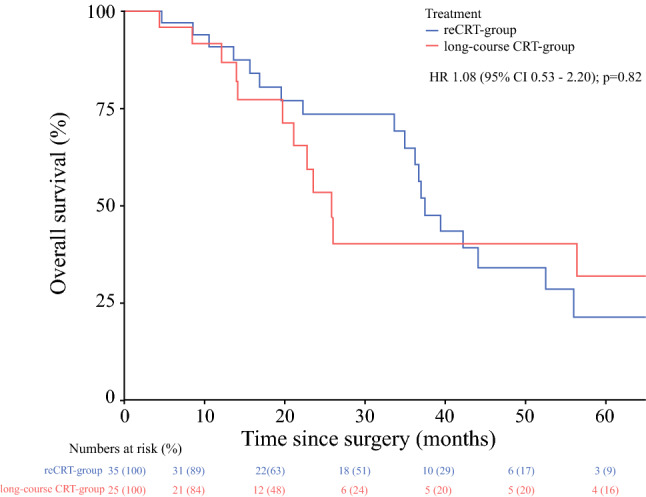
Overall survival. *CRT* chemoradiotherapy, *HR* hazard ratio, *CI* confidence interval

**Fig. 2:**
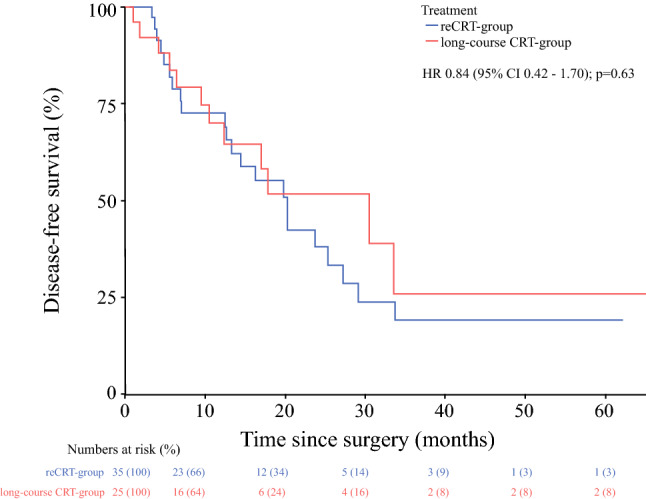
Disease-free survival. *CRT* chemoradiotherapy, *HR* hazard ratio, *CI* confidence interval

**Fig. 3:**
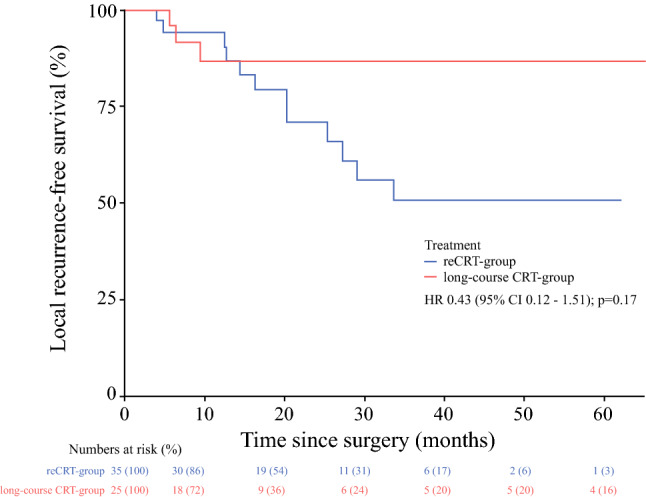
Local recurrence-free survival. *CRT* chemoradiotherapy, *HR* hazard ratio, *CI* confidence interval

The median follow-up in the reCRT group was 53 months (IQR 25–53), and 38 months (IQR 17–65) in the long-course CRT group. The 3- and 5-year OS for the reCRT group was 64.9% and 21.3%, respectively, and in the long-course CRT group, 3- and 5-year OS was 40.1% and 32.1%, respectively (*p* = 0.82) (Fig. 1). The median interval between RRC and re-recurrent disease was 13 months (IQR 5–20). Patients in the reCRT group had 3- and 5-year DFS rates of 19.0% and 19.0%, respectively, and in the long-course CRT group, 3- and 5-year DFS was 25.8% and 25.8%, respectively (*p* = 0.63) (Fig. 2). In the reCRT group, LRFS was 50.7% and 50.7% 3 and 5 years after surgery, and 86.5% and 86.5% in the long-course CRT group, respectively (*p* = 0.17) (Fig. 3). The use of IOBT does not influence the risk of developing local re-recurrent disease (*p* = 0.44) [electronic supplementary Fig. S1].

## Discussion

The primary purpose of this study was to evaluate toxicity and oncological outcomes in patients with RRC after reCRT compared with long-course CRT.

No acute grade IV or V toxicities were reported. Acute grade III toxicity occurred in two patients in the reCRT group (5.7%, diarrhea and obstruction). Studies with the same re-irradiation regimens (mostly combined with chemotherapy) showed a higher incidence of grade III–V acute toxicity of 6–9%.^14^^,^^28^ In both these studies, toxicity was also scored retrospectively, which does not explain the difference in acute toxicity. Furthermore, after a higher median re-irradiation dose (34.5–50 Gy), mostly combined with chemotherapy, grade III–IV toxicities occurred in 4–9% of cases.^12^^,^^15^^,^^29^ A recently published meta-analysis showed 11.7% acute grade III or higher toxicity after re-irradiation (2 prospective studies of 11 included studies).^9^ Patients in the long-course CRT group in our study reported significantly more skin toxicity (*p* = 0.040). This could be explained by the fact that patients in the long-course CRT group received much more capecitabine, which is known for its skin toxicity.^30^

We have shown 3-year OS rates in the reCRT group of 64.9%; however, in previously conducted studies, using the same regimen, the 3-year OS rates varied between 49% and 66%.^14^^,^^15^^,^^18^ A meta-analysis, in which the radiotherapy doses ranged between 16 and 40.8 Gy, found a 3-year OS rate of 51.7%.^9^ Regarding radicality, pCR, OS, DFS, and LRFS, we did not find any significant differences between the two groups, which suggests that re-irradiation is just as effective as irradiation. Furthermore, we demonstrated 3- and 5-year LRFS rates of 50.7% and 50.7% in the reCRT group, while an additional study with a higher median re-irradiation dose showed 3- and 5-year local control of 46.6% and 38.8%, respectively.^12^ This suggests that a higher re-irradiation dose does not correlate with better local control. In addition, the study by Alberda et al., in which the same treatment strategy was used, demonstrated 3-year local control of 48.6%, which is comparable with our study.^18^ In the case of re-irradiation, radiotherapy response did occur in this group; however, there was the possibility of selection of radiotherapy-resistant tumors that could relapse. In the long-course CRT group, patients had initially relatively low-risk primary tumors (not requiring radiotherapy) that relapsed unexpectedly, which is probably a negative risk factor. In contrast, the reCRT group included patients with intermediate- or high-risk tumors who required radiotherapy as part of their initial treatment, and in which a recurrence could be expected. Therefore, the selection of tumors with differences in biological behavior might have been different.

The location of the recurrence was most often lateral in the reCRT group (34.3%) and at the anastomosis in the long-course CRT group (50.0%). After all, patients in the reCRT group were previously irradiated because the primary tumor was a locally advanced tumor that often recurs at the borders of the radiotherapy field. This has also been confirmed by the Dutch TME trial demonstrating that lateral recurrences occurred in 25% of patients who received radiotherapy (5 × 5 Gy followed by immediate surgery). In addition, that study also showed that lateral recurrences are associated with poor prognosis.^23^ This is because it is more difficult to achieve an R0 resection at the lateral resection borders,^1^ which may explain the significant difference in the R1 resection rate between the two groups in our study. However, in the reCRT group, it was found there was no difference in survival between lateral recurrences and recurrences at other places (*p* = 0.14, data not shown). Of those patients who did not receive radiotherapy in the TME trial, local recurrences at the site of the anastomosis occurred in 24.4%, which is much lower than the 50.0% found in our study. Furthermore, the TME study showed that preoperative radiotherapy reduced the anastomotic recurrence rate,^23^ which explains the lower number of anastomotic recurrences in the reCRT group in our study (17.1%).

Although the R1 resection rate was higher in the reCRT group (51.4% vs. 24.0%), this is possibly not explained by the lower radiation dose in the re-irradiation group. After all, patients in the reCRT group were previously irradiated because the primary tumor was a locally advanced tumor that often recurs at the borders of the radiotherapy field, which makes the resection more difficult.^31^ Perhaps re-irradiation more often results in non-response. Therefore downsizing of the tumor will not occur, which in turn may hamper a radical resection. An R0 resection was seen in 42.9% of patients in the reCRT group. In studies using a comparable re-irradiation schedule, the R0 resection rate varied between 46% and 70%, while the R0 resection rate was 35.6% after a higher median re-irradiation dose of 40.8 Gy.^12^^,^^14^^,^^18^^,^^28^ This demonstrated that there is no association between the median re-irradiation doses and the R0 resection rate. The R0 resection rate after long-course CRT in the study by Alberda et al. was 63%, which is comparable with the 68% found in our study.^18^

IOBT was significantly more often used in the reCRT group (40% vs. 16%), which we believe is because it is the last resort in re-irradiated RRC patients, given the fact that patients in the long-course CRT group are still able to receive the re-irradiation schedule in case of re-recurrent disease. The higher number of R1 resections in the reCRT group could also be explained by the use of IOBT, since IOBT is only performed in cases an R1 resection is suspected. However, the use of IOBT does not influence the cumulative probability of developing local re-recurrent disease (*p* = 0.44). The decision to perform IOBT was at the discretion of the surgeon, together with the radiation oncologist. The accuracy to correctly judge the radicality of resection was accompanied by a sensitivity of 80.0% in the reCRT group and 88.2% in the long-course CRT group. This difference could be explained by fibrosis, which could be more prominent in the reCRT group due to radiotherapy. Fibrosis makes a resection more difficult, which could also be the reason why frozen section pathology was more often performed in the reCRT group (45.7% vs. 8%).

In the study by Valentini et al., which had a higher re-irradiation dose (40.8 Gy), the pCR rate was approximately 8.5%;^12^ however, in the study by Alberda et al., which used the same re-irradiation regimen as our study, the pCR rate was 4%,^18^ which is approximately twice as low as in our study (8.6%). The accumulated pCR rate in our study was 10%, which is lower than the 19% found in the study by Voogt et al.^32^ In this retrospective study, patients received induction chemotherapy consisting of three cycles of CAPOX or four cycles of FOLFOX followed by the same long-course CRT or re-irradiation schedules as in our study.^32^ This almost double pCR rate suggests that the use of oxaliplatin may result in more downstaging and downsizing of the tumor, an hypothesis that is supported by the fact that the R0 resection rate in the total group was higher (63% vs. 53%) in the study by Voogt et al.^32^

Fibrosis often occurs after neoadjuvant treatment of RRC. At restaging with MRI, it is challenging to distinguish fibrosis from tumor tissue, and thus it is difficult to determine if a cCR occurred. This could be the reason for the significant difference in cCR on MRI between the two groups in favor of the long-course CRT group. As these radiotherapy-naïve patients received an overall lower radiotherapy dose to the pelvis compared with the reCRT group, likely results in less fibrosis. Therefore, it could be that patients in the reCRT group are more often understaged at restaging, whereas patients in the long-course CRT group are overstaged. Another explanation is that recurrent disease could be accompanied by negative selection, with a lower chance of a complete response. It is therefore risky to use a wait-and-watch (W&W) strategy. The oncological outcomes after a W&W strategy are unknown in RRC. In our study, an MRI-based cCR was accomplished in 2.9% of reCRT patients, while the pCR rate in these patients was 8.6%. Therefore, the sensitivity and specificity of MRI-based cCR were 33.3% and 100% in the reCRT group, respectively. In addition, the degree of (pre-existing) fibrosis related to the previous radiotherapy and surgery possibly also led to the difference in non-organ bound resections (31.4% vs. 12.0% in the reCRT and long-course CRT groups, respectively).

Depending on the time interval, normal tissue possibly recovers after radiotherapy. In cases where the interval is ≥ 1 year, it is considered safe to re-irradiate patients with a dose of 30 Gy.^13^^,^^15^ In the reCRT group, we showed a median interval between prior radiation and the onset of re-irradiation of 29 months. Based on the absence of high-grade toxicities in the current study and the limited toxicity reported in the studies by Valentini et al., Das et al., and Koom et al.,^12^^,^^15^^,^^29^ a higher re-irradiation dose (30–40 Gy) could be considered if the interval is ≥ 1 year.

The treatment of RCC has become more sufficient during the last decades. Earlier, we reported an historical cohort of patients from our center, revealing a 5-year OS rate of 19%^33^ to 32% in the current study. LRFS increased from 30% to 39% 5 years after surgery to 86.5% in patients who received long-course CRT.^33^^,^^34^ Moreover, in the study by Reerink et al., the distant metastasis rate after the treatment of RRC decreased from 57.5% to 40% in the present study in the case of long-course CRT.^33^ There are some possible explanations for the differences. First, there were differences in treatment characteristics; only 12.5% of patients in the study by Reerink et al. received concurrent chemotherapy and some patients received postoperative radiotherapy.^33^ Second, MRIs were not performed in the previously conducted studies.^33^^,^^34^ Third, there was often no standardized follow-up.^33^ Finally, the quality of CT scans has increased over the last decades, possibly resulting in better selection.

In the literature, there is as yet no consensus on the best treatment for RRC patients who received (chemo)radiotherapy for their primary tumor; the radiotherapy doses for RRC ranged from 15.0 to 49.2 Gy. In addition, chemotherapy is not always prescribed.^35^ This makes it harder to compare the results of our study with the currently available literature. In addition, as in our study, most literature contains heterogeneous data. Other limitations are the retrospective nature of the study, which may have resulted in an underestimation of the treatment-related toxicities and the occurrence of a small sample size, however recurrence of rectal cancer is relatively rare (recurrence rate of 5–9%). Therefore, we would recommend an (inter)national prospective cohort study to consider outcomes and toxicity.

## Conclusion

Re-irradiation is well tolerated and is associated with low toxicity and comparable oncological outcomes. Although re-irradiation was associated with lower cCR, there was no difference in pCR. In the re-irradiation group, an irradical resection was more often achieved (not significant), which may be due to the more challenging locations of the recurrence compared with CRT-naïve patients. We conclude that it is safe and feasible to re-irradiate RRC patients.

## Supplementary Information

Below is the link to the electronic supplementary material.

Supplementary file1 (DOCX 170 KB)
