# Supplementary material for: Re-Irradiation in Patients with Recurrent Rectal Cancer is Safe and Feasible
Source: Ann Surg Oncol. 2021 May 22;28(9):5194–204. doi: 10.1245/s10434-021-10070-6 (PMC8349344; doi:10.1245/s10434-021-10070-6)
Supplement: Supplementary file 1 — Supplementary file1 (DOCX 170 KB) [file 10434_2021_10070_MOESM1_ESM.docx]

online SUPPLEMENT

Table of Content

|  |  | Page |
| --- | --- | --- |
| Figure S1 | Cumulative probability of developing local re-recurrent disease | 2 |
| Table S1 | Participating institutes and collaborative investigators | 3 |

| 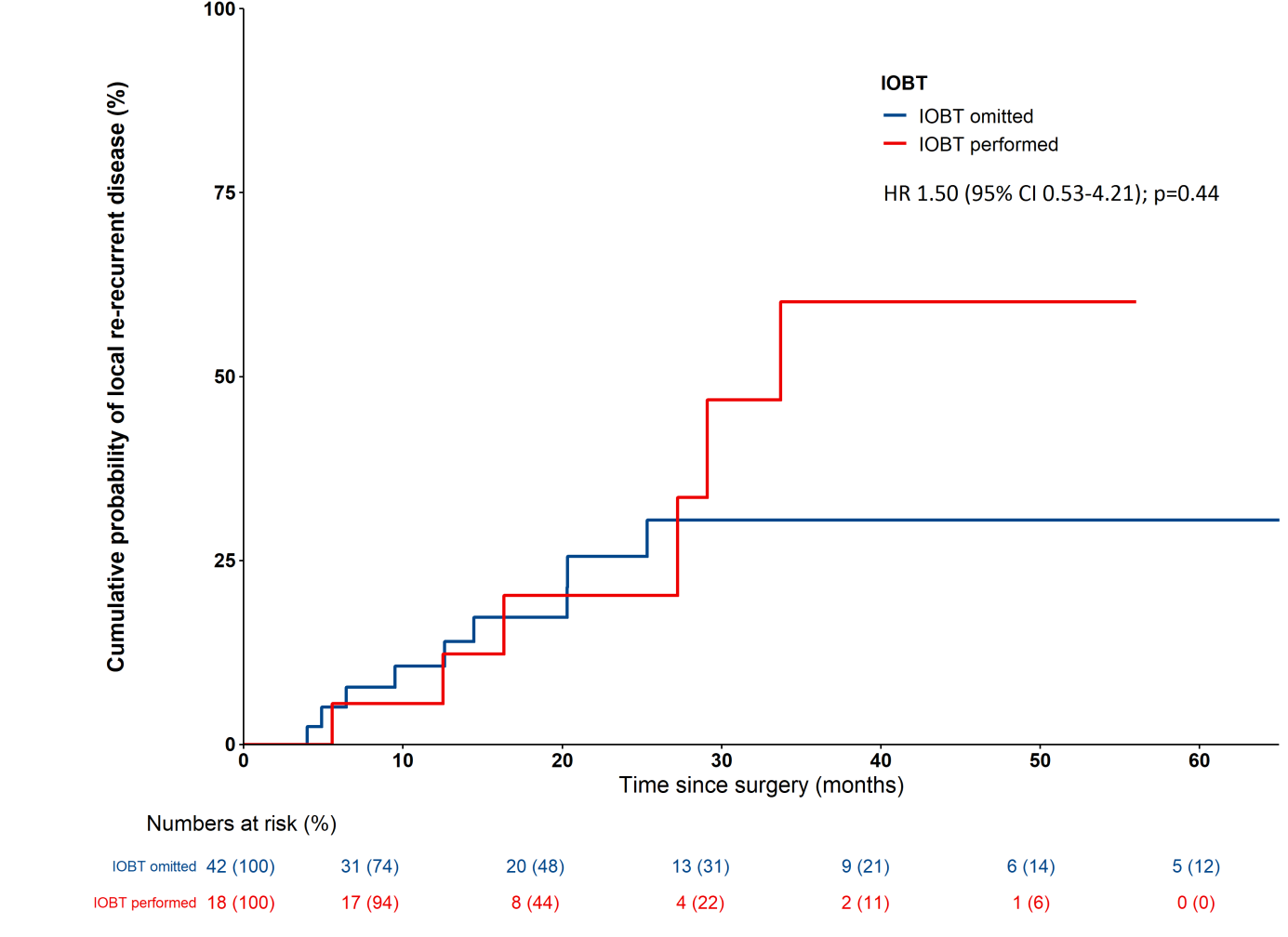 |
| --- |
| **Figure S1**  Cumulative probability of developing local re-recurrent disease |

| **Table S1**  Participating institutes and collaborative investigators | | | |
| --- | --- | --- | --- |
| Location | Institute | Department | Investigator |
| Almelo | Ziekenhuisgroep Twente | Department of surgery | I. F. Faneyte |
| Assen | Wilhelmina Hospital | Department of surgery | S. T. van Vugt |
| Deventer | Deventer Hospital | Department of surgery | R. J. I. Bosker |
| Drachten | Nij Smellinghe Hospital | Department of surgery | I. T. A. Pereboom |
| Enschede | Medisch Spectrum Twente | Department of surgery | P. Steenvoorde |
| Emmen | Scheper Hospital | Department of surgery | R. A. Schasfoort |
| Groningen | Martini Hospital | Department of surgery | P. C. Baas |
| Hardenberg | Röpcke-Zweers Hospital | Department of surgery | M. F. Lutke-Holzik |
| Heerenveen | Tjongerschans Hospital | Department of surgery | F. Wit |
| Hoogeveen | Bethesda Hospital | Department of surgery | F. W. H. Kloppenburg |
| Leeuwarden | Medical Center Leeuwarden | Department of surgery | M. A. Kaijser |
| Meppel | Diaconessenhuis Meppel | Department of surgery | F. N. L. Versluijs-Ossewaarde |
| Scheemda | Ommelander ziekenhuis Groningen | Department of surgery | D. P. de Vries |
| Sneek | Antonius Hospital | Department of surgery | D. A. Hess |
| Winterswijk | Streekziekenhuis Koningin Beatrix | Department of surgery | B. Inberg |
| Zwolle | Isala Klinieken | Department of surgery | A. D. van Dalsen |
